# Changes in zinc-α2-glycoprotein (ZAG) plasma concentrations pre and post Roux-En-Y gastric bypass surgery (RYGB) or a very low calorie (VLCD) diet in clinically severe obese patients: Preliminary Study

**DOI:** 10.15761/IOD.1000170

**Published:** 2017-03-06

**Authors:** Kyle W Morse, Nerys M Astbury, Alexandra Walczyszyn, Sami A Hashim, Allan Geliebter

**Affiliations:** 1 Weill Cornell Medical College, New York, NY, USA; 2 Department of Psychiatry, Mt Sinai St. Luke’s Hospital, New York, NY, USA

**Keywords:** obesity surgery, ZAG, weight loss, lipolysis, VLCD

## Abstract

The purpose of this preliminary study was to investigate changes in plasma concentrations of zinc-α2-glycoprotein (ZAG), a lipid mobilizing hormone, in obese subjects following Roux-En-Y Gastric Bypass (RYGB) surgery or a very low calorie diet (VLCD). Fasting blood concentrations and anthropometric measurements were measured pre and 12 weeks post intervention. 14 healthy, obese individuals underwent either RYGB (N=6) surgery or a VLCD (N=8). Body composition and fasting plasma ZAG concentrations were measured at baseline (pre) and 12 weeks post intervention (post). At pre-intervention baseline, there was no difference in plasma ZAG between the two intervention groups. Post-intervention, there was a significant overall reduction (F(1,11) = 32.8, *p*<0.001) in plasma ZAG, which was significant only within the RYGB group from pre to post intervention (33.2 ± 5.7 μg/ml to 26.7 ± 4.8 μg/ml (*p*<0.015)) and significantly greater than the change within the VLCD group. The change in ZAG was inversely correlated across groups with BMI reduction (r= −0.60, *p*<0.05), % body fat reduction (r= −0.68, *p*<0.015), reduction in weight (r= −0.58, *p*<0.05), and % weight loss (r= −0.70, *p*<0.05). Overall, subjects who underwent RYGB or VLCD had a significant reduction in plasma ZAG. This reduction was significant within the RYGB group alone, who lost a larger amount of weight than the VLCD group, which suggests that ZAG may have a protective effect during marked weight loss.

## Introduction

Over one third of the United States population is obese (BMI >30 kg/m^2^) [1]. Bariatric surgery is a relatively effective long-term treatment for obesity [2] especially Roux-en-Y Gastric Bypass (RYGB) [3]. Weight loss following RYGB is due to restriction and possibly malabsorption, [4] but the specific biological mechanisms are not well understood.

Zinc-α2-glycoprotein (ZAG) is a 41-kDa protein, which was first isolated from human plasma in 1961 [5]. ZAG shares the same structure as a lipid mobilizing factor [6] that is over-expressed in many different cancer types and has been associated with the rapid weight loss in cancer patients [7]. ZAG is secreted by adipocytes [8] in the white adipose tissue (WAT) and utilizes the G-protein coupled β3-adrenoreceptor to activate adenylate cyclase, form cyclic AMP (cAMP), activate protein kinase A (PKA), and induce lipolysis [9,10]. Russell *et al.* [11] found that ZAG induced both adipose triglyceride lipase (ATGL) and hormone sensitive lipase (HSL) expression. HSL is also regulated through the cAMP pathway and PKA [12]. The up-regulation of HSL results in increased lipolysis. ZAG also up-regulates the amount of uncoupling protein 1 (UCP-1) [13] in brown adipose tissue, which may increase lipid utilization to produce heat.

Plasma levels of both ZAG and ZAG mRNA are significantly lower in obese patients than lean controls, [14] which may reduce lipid mobilization and utilization in the obese [15]. ZAG is inversely correlated with body mass index (BMI), body weight, body fat percentage, and fat mass [16,17]. Genetically obese ob/ob mice that were given ZAG intravenously or intraperitoneally, lost weight [10,18] and body fat [11]. In this study, we examined plasma ZAG concentrations pre and post two weight loss interventions, RYGB or a very low calorie diet (VLCD), and we hypothesized that ZAG would increase with weight loss.

## Materials and methods

### Design

During the period from December 30^th^, 2010 through October 9^th^, 2012, 14 severely obese subjects (BMI = 43.34 ± 3.32 kg/m^2^) were recruited from individuals seeking weight loss treatment at St. Luke’s-Roosevelt Hospital, New York, NY from a larger study. Candidates for RYGB at the Center for Bariatric Surgery and Metabolic Disease were enrolled in the surgical group (N=6, male/female: 0/6), and candidates, matched for BMI were enrolled in the VLCD group (N=8, male/female: 2/6). The IRB at St. Luke’s-Roosevelt Hospital Center and at Columbia University Medical Center approved the study, and the participants gave informed consent. The study was registered with Clinical Trials Registry number, NCT01583725.

### Participants

Candidates were eligible if they were between ages 18 and 65, had a BMI between 35 and 50, and were approved to undergo RYGB or receive a VLCD. Candidates with current use of prescribed medications, smokers, and those exercising greater than 5 hours/week were excluded. Those with previous abdominal surgery or current significant health problems, i.e., endocrine disorders, cancer, heart, kidney, thyroid, gastrointestinal, liver disease, and anemia (Hct <30%) also were excluded.

### Study protocol

All subjects were assessed pre- and 12-week post-intervention. The VLCD provided 1012 kcal (0.86 kcal/mL, 45% protein, 38% carbohydrate, 17% fat by energy) per day from the combination of five packets of Procal-100 (R-Kane, Pennsauken, New Jersey, USA) and 1182 mL of low fat (1% fat content) milk. Subjects were placed on this formula diet for 12 weeks, and attended individual weekly nutritional and behavioral sessions and received packets at no cost.

Prior to each experimental visit, participants were asked to consume a 500 kcal liquid meal (1 kcal/mL, 18% protein, 40% carbohydrate, 42% fat) of Glytrol (Nestle Health Sciences, Lutry, Switzerland) at 8 PM during the evening and to consume no other foods or drinks apart from water until they arrived at the laboratory at 8 AM the following morning. Upon arrival, height was measured to the nearest cm with a stadiometer (Detecto Scales Inc, Brooklyn, NY). Weight and body fat mass were measured to the nearest 0.1 kg by bioelectrical impedance (TBF 300a; Tanita Corporation, Itabashi-Ku, Tokyo, Japan). A blood sample was collected from the median cubital vein in the non-dominant arm by venipuncture and placed in EDTA tubes, and cold centrifuged (3000 RPM) at 4°C for 15 min to obtain plasma samples. Samples were stored at −80°C until assayed for ZAG with an ELISA kit (BioVendor Laboratories, Brno, Czech Republic., with an intra-assay variation of 0.60–3.89%.

### Statistical analysis

SPSS version 22 (IBM, Armonk, NY, USA) was used for data analysis. Homogeneity of variance was tested by Levine’s test. Possible covariates such as age, gender, BMI reduction, fat loss, weight loss, percent body fat loss, and time between assessments were tested using univariate ANCOVA, and only age was a significant covariate for the change in ZAG. When the overall F from the ANCOVA was significant, post-hoc t tests were used to identify specific differences. Scatterplots and regression lines were plotted, and Pearson correlation coefficients were calculated between ZAG, body weight, % weight loss, % fat loss, and BMI. Two-tailed p values <0.05 were considered significant. A power analysis was not performed due to participants being a subset from a subject pool participating in a larger study.

## Results

### Subject characteristics

There were no significant baseline differences between groups other than fat mass (Table 1). Both groups showed significant changes in BMI, weight, fat mass, and fat percentage pre to post intervention. Weight decreased more in the RYGB group, 27.3 ± 3.5 kg, than in the VLCD group, 13.4 ± 1.3 kg (t(12) = 6.3, *p* = 0.001). Similarly, % weight loss was significantly greater in the RYGB group, 21.2 ± 1.7 %, than in the VLCD group, 11.2 ± 1.0 % (t(12) = 8.0, *p* < 0.001) as was loss of fat mass 21.3 ± 4.4 kg vs. 8.7 ± 1.8 kg (t(10) = 5.3, *p* < 0.05) and reduction in % body fat, 7.9 ± 1.4 % vs., 2.8 ± 1.3 % (t(10)= 9.0, *p*<0.05). At baseline, there were no significant correlations between ZAG and BMI, body weight, fat mass, % body fat or age.

### Effect of intervention type on plasma ZAG level

**Figure 1:**
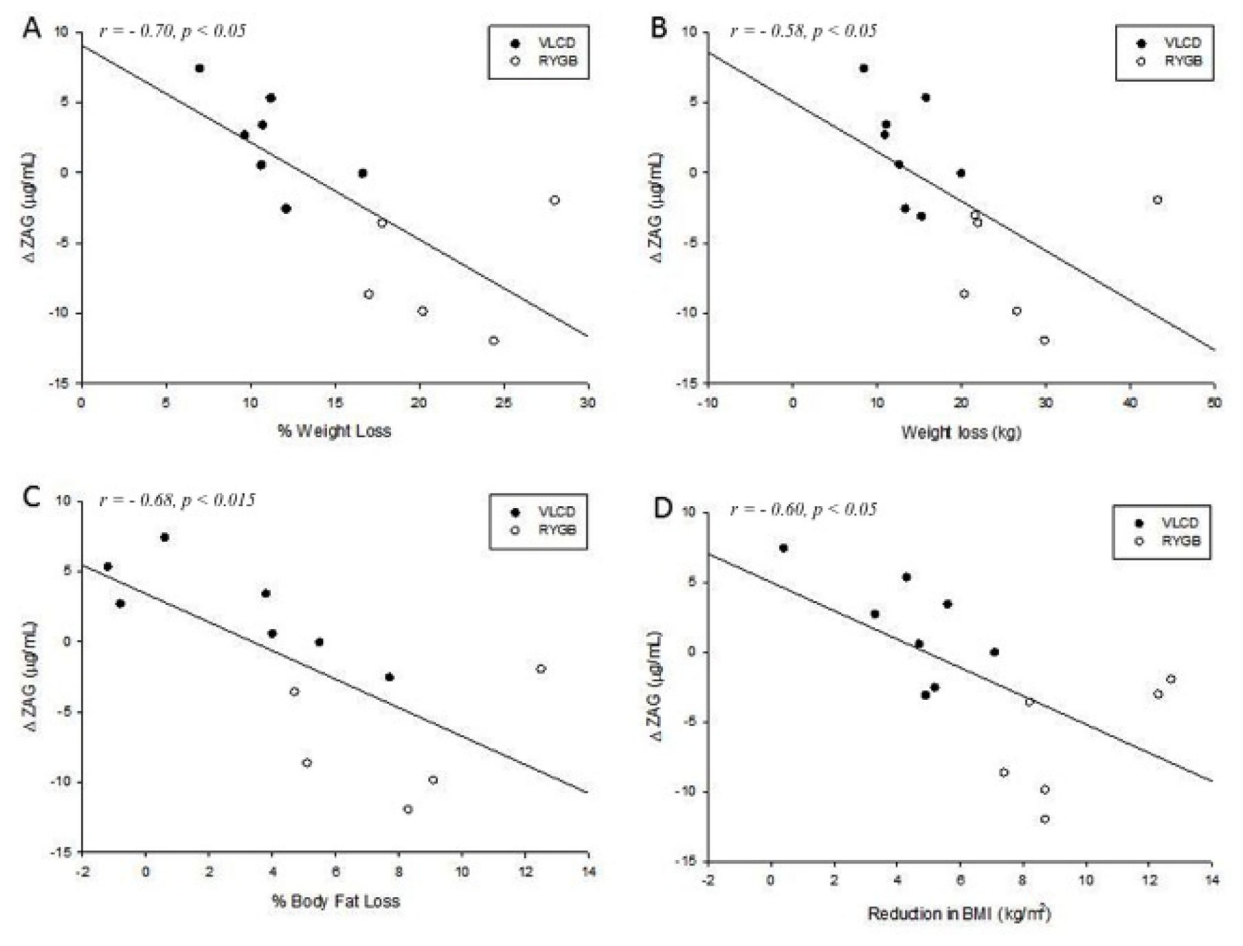
Scatter plots and regression lines for the change in (A) plasma ZAG concentrations and (B) % weight loss, (C) % body fat loss, and (D) reduction in BMI in subjects who underwent RYGB or VLCD. Across groups, change in plasma ZAG concentrations was inversely correlated with % weight loss (r= −0.70, *p*<0.05), amount of weight loss (r= −0.58, *p*<0.05), % body fat loss (r= −0.68, *p*<0.015), and reduction in BMI (r= −0.60, *p*<0.05). Note that above a certain threshold of weight loss, ZAG begins to decrease for those in the VLCD group.

A repeated measures ANCOVA with age as a covariate showed a significant time x treatment interaction (F(1,11)=32.8, *p*<0.001). A post hoc t test showed a significant decrease in plasma ZAG within the RYGB group (33.2 ± 2.3 μg/ml to 26.7 ± 1.9 μg/ml (*p*<0.015)), and a non-significant increase within the VLCD group (33.1 ± 2.1 μg/ml to 34.9 ± 2.0 μg/ml) (*p=*0.23, ns), resulting in a significant difference between groups (*p*<0.01). Further analysis showed that the change in ZAG was negatively correlated across groups with BMI reduction (r=−0.60, *p* <0.05), % body fat reduction (r= −0.68, *p*<0.015), weight loss (r= −0.58, *p*<0.05) and % weight loss (r= −0.70, *p*<0.05) (Figure 1). Additionally, the change in plasma ZAG within the VLCD group was negatively correlated with the body fat mass reduction (r= −0.84, *p*<0.05 and % body fat reduction (r= −0.82, *p*<0.05). A univariate ANCOVA controlling for age showed that the difference in ZAG concentration between groups could not be accounted for by change in fat mass, weight loss, time between assessments, or BMI, when entered individually as covariates.

## Discussion

In this study, we examined ZAG plasma concentrations in two groups of severely obese subjects undergoing two different weight loss interventions. Across groups, there was a significant reduction in ZAG, but the reduction in ZAG was significant within the RYGB group but not in the VLCD group.

In previous studies, ZAG correlated with BMI and other body composition measures. Selva *et al.* [14] reported that ZAG negatively correlated with BMI while Gong *et al.* [17] found ZAG negatively correlated with BMI, waist circumference, hip circumference, percentage of body fat, and fat mass. We did not find any significant correlations between baseline body composition and ZAG. These findings may be due to our relatively small sample within a restricted high BMI range. Age was found to be a significant covariate for the change in ZAG following the intervention although age did not correlate significantly with ZAG at baseline and did not differ between groups. Hirai *et al.* [10] found that *in vitro* administration of high concentrations of human lipid mobilizing factor (derived from the urine of cancer patients) to human adipocytes, led to down-regulation of lipolysis. This down-regulation may help achieve oxidative balance by utilizing fatty acids to meet the body’s energy demands.

Those in the RYGB group had a significant decrease in ZAG, whereas in the VLCD group, only those who lost a substantial amount had a decrease in ZAG. There may be a threshold at which ZAG is down-regulated, as those undergoing RYGB lost more weight than those receiving VLCD. Previous studies found that VLCD’s led to reduced adipocyte size, [19] and this size reduction may also affect adipokine secretion [20]. Rossmeislova *et al.* [19] observed a reduction of adipokines and cytokines following VLCD and low calorie diets but did not examine ZAG. Ge *et al.* [21] studied ZAG following weight loss and weight loss with aerobic exercise over a six-month time period. They showed a non-significant increase in ZAG in both groups with a 8% reduction in body weight in both groups [21]. These results are similar to our VLCD group, which also showed a non-significant increase in ZAG following a 11.2% decrease in weight.

RYGB results in markedly lower food intake, which leads to rapid weight loss within the first few months resulting in a semi-starvation state. However, long-term energy needs still need to be met, and ZAG may be down-regulated to protect adipocyte function during this period. Ceperuelo-Mallafre *et al.* [22] treated adipocytes with ZAG, which resulted in glucose intake by upregulation of GLUT-1 and GLUT-4 receptors. Interestingly, this study also found a paradoxical inhibition of insulin though PP2A activation [22]. While we cannot correlate ZAG to insulin levels in our study, it is possible that the reduction in ZAG following RYGB may result in decreased insulin resistance and investigating this relationship would be an area for future research. A better understanding of the protective mechanisms involved in weight loss could aid in developing more effective long-term weight loss treatments. A limitation of this study is the small number of subjects, and larger additional studies are needed. Another limitation is that other hormones involved in the regulation of metabolism were not measured and compared with ZAG. In summary, we have shown in a preliminary study that ZAG plasma levels change following weight loss intervention, with a significant decrease of plasma ZAG in RYGB patients.

## Figures and Tables

**Table 1:** Body composition parameters of study participants from pre-intervention (Pre) to 12 weeks post-intervention (Post) in subjects who underwent RYGB surgery or a VLCD. Values represent means ± standard deviation.

	RYGB		VLCD	
	*Pre*	*Post*	*Pre*	*Post*
Age	44.2 ± 11.7		41.0 ± 9.5	
BMI (kg/m^2^)	44.8 ± 3.3	35.1 ± 4.0***	42.3 ± 3.2	37.8 ± 4.6***
Weight (kg)	127.0 ± 15.0	99.8 ± 8.1***	119.9 ± 12.2	106.5 ± 11.2***
Fat Mass (kg)	66.5 ± 11.4	45.2 ± 4.4**	54.1 ± 5.9^a^	45.5 ± 6.8**
Fat Percentage (%)	52.3 ± 1.7	44.3 ± 3.7**	46.3 ± 6.9	43.5 ± 6.7
ZAG (μg/ml)	33.2 ± 5.7	26.7 ± 4.8*	33.1 ± 6.1	34.9 ± 5.6^a^

* *p* <0.05 change from baseline condition

** *p*< 0.01 change from baseline condition

*** *p* ≤ 0.001 change from baseline condition

a *p*< 0.05 difference between change in ZAG following RYGB and VLCD
